# Factors Associated with Live Birth After Placenta-Derived Mesenchymal Stromal Cell Therapy in Women with Recurrent Intrauterine Adhesions and Thin Endometrium

**DOI:** 10.3390/life16060871

**Published:** 2026-05-22

**Authors:** Tabeeva Giuzial, Silachev Denis, Asaturova Aleksandra, Shevtsova Yulya, Yurin Alexander, Popov Konstantin, Pronin Stanislav, Korshunov Alexey, Dzhabiev Alan, Smetnik Antonina, Marchenko Larisa, Chernukha Galina, Sukhikh Gennady

**Affiliations:** 1 V.I. Kulakov National Medical Research Center for Obstetrics, Gynecology and Perinatology, Ministry of Healthcare of Russian Federation, 117997 Moscow, Russia; a.asaturova@gmail.com (A.A.); y_shevtsova@oparina4.ru (S.Y.); a_yurin@oparina4.ru (Y.A.); k_popov@oparina4.ru (P.K.); s_pronin@oparina4.ru (P.S.); a_korshunov@oparina4.ru (K.A.); a_dzhabiev@oparina4.ru (D.A.); a_smetnik@oparina4.ru (S.A.); l_marchenko@oparina4.ru (M.L.); g_chernukha@oparina4.ru (C.G.); g_sukhikh@oparina4.ru (S.G.); 2 Department of Functional Biochemistry of Biopolymers, A.N. Belozersky Research Institute of Physico-Chemical Biology, Moscow State University, 119992 Moscow, Russia

**Keywords:** placenta-derived mesenchymal stromal cells, cell therapy, intrauterine adhesions, thin endometrium, endometrial regeneration, regenerative medicine

## Abstract

Recurrent intrauterine adhesions (IUA) and refractory thin endometrium are associated with impaired endometrial regeneration, reduced implantation, and poor live birth outcomes. Regenerative therapy using mesenchymal stromal cells (MSCs) has shown promising results; however, factors associated with reproductive success remain unclear. In this prospective, single-centre, single-arm uncontrolled observational study, 35 women with recurrent IUA and thin endometrium (<7 mm) unresponsive to standard surgical and hormonal therapy received combined subendometrial and systemic administration of placenta-derived MSCs. The primary endpoint was live birth. Secondary endpoints included clinical pregnancy rate, time to pregnancy, endometrial thickness changes, uterine blood flow (resistance index, RI), and anti-Müllerian hormone (AMH) levels. Univariable logistic regression was performed to identify factors associated with live birth. Clinical pregnancy occurred in 13/35 patients (37.1%), and live birth was achieved in 11/35 (31.4%). Median time to pregnancy was 7 (5–8) months. Shorter duration of infertility or prior pregnancy loss (OR 1.55 per year; 95% CI 1.10–2.57), AFS stage I adhesions (OR 6.8; 95% CI 1.1–42; *p* = 0.04), lower baseline RI in uterine, arcuate and radial arteries, and higher baseline AMH (OR 2.59 per doubling; 95% CI 1.15–6.89) were significantly associated with live birth. Endometrial thickness increased after therapy but was not significantly associated with live birth. No severe adverse events were observed. Placenta-derived MSC therapy was followed by live birth in 31.4% of women with recurrent IUA and refractory thin endometrium. A shorter duration of reproductive disorders, less severe adhesions, lower baseline RI in uterine, arcuate and radial arteries, and higher AMH levels were associated with live birth after treatment and may help identify patients with a more favourable reproductive prognosis in future controlled studies.

## 1. Introduction

Impaired endometrial regeneration, resulting in intrauterine adhesions (IUA) and refractory thin endometrium, remains one of the most challenging conditions in reproductive medicine [1]. Both conditions are associated with reduced implantation rates, lower clinical pregnancy and live birth rates, and an increased risk of miscarriage [2]. Despite advances in hysteroscopic surgery and assisted reproductive technologies, restoration of functional endometrial receptivity after severe endometrial damage remains limited [3,4].

Hysteroscopic adhesiolysis is considered the standard treatment for IUA [5,6]; however, recurrence rates remain high, particularly in moderate and severe cases [2,4]. Even after anatomical restoration of the uterine cavity, persistent stromal fibrosis, impaired angiogenesis, and defective endometrial proliferation frequently compromise reproductive outcomes [2,7]. Thin endometrium, commonly defined as an endometrial thickness of less than 7 mm during the implantation window, represents a clinical manifestation of insufficient regenerative capacity and is strongly associated with implantation failure and poor live birth rates [8,9,10].

Conventional therapeutic approaches—including estrogen therapy, intrauterine devices, platelet-rich plasma, growth factors, and vasodilators—have shown inconsistent results, especially in women with recurrent adhesions and refractory endometrium [5,11,12,13,14]. Therefore, regenerative strategies targeting stromal remodeling, angiogenesis, and immunomodulation are increasingly explored [15,16,17,18].

Mesenchymal stromal cells (MSCs) possess anti-fibrotic, pro-angiogenic, immunomodulatory, and trophic properties, mediated largely through paracrine signaling and extracellular vesicle secretion [19]. Preclinical studies demonstrate that MSC therapy promotes endometrial regeneration, enhances vascularisation, reduces fibrosis, and improves receptivity markers [17,18]. Early clinical reports suggest potential benefits in women with severe endometrial damage; however, the available evidence is limited to small pilot studies, and robust data on reproductive outcomes remain scarce [20,21,22].

While most clinical studies on intrauterine adhesions have focused on MSCs derived from bone marrow, adipose tissue, umbilical cord, menstrual blood and endometrial tissue, placenta-derived MSCs offer several potential advantages, including higher proliferative capacity, lower immunogenicity, and more pronounced paracrine activity. Additionally, placenta-derived MSCs are highly scalable for clinical applications, making them an attractive therapeutic source [23,24,25].

Nevertheless, several critical clinical questions remain unresolved, including which patients are most likely to benefit from cell therapy, which biological or functional parameters are associated with treatment success, and whether restoration of endometrial structure ultimately translates into live birth.

Identifying factors associated with reproductive success after regenerative therapy is essential for patient selection and the development of individualized treatment strategies. In particular, vascular parameters, ovarian reserve markers, and the severity of intrauterine adhesions may influence therapeutic response but have not been systematically evaluated in this context.

Although previous studies have demonstrated improvements in endometrial thickness and reproductive outcomes following MSC therapy, factors associated with treatment response remain poorly understood [19,21,22]. In particular, vascular parameters reflecting endometrial perfusion have not been systematically evaluated for their association with treatment response in women with intrauterine adhesions.

The aim of this single-arm uncontrolled observational study was to evaluate reproductive outcomes following placenta-derived MSC therapy in women with recurrent intrauterine adhesions and refractory thin endometrium, and to identify clinical, ultrasonographic, hormonal, and morphological factors associated with live birth.

## 2. Materials and Methods

### 2.1. Study Design

This prospective, single-centre, single-arm uncontrolled observational study was conducted between January 2023 and December 2025 at the V.I. Kulakov National Medical Research Centre for Obstetrics, Gynaecology and Perinatology of the Ministry of Health of the Russian Federation (Moscow, Russia). The primary objective was to evaluate reproductive outcomes and identify factors associated with live birth following therapy with placenta-derived MSCs in women with recurrent IUA and refractory thin endometrium.

The study used a within-subject longitudinal design. All participants underwent combined subendometrial and systemic administration of MSCs after failure of standard surgical and hormonal therapies. Subendometrial MSC administration was performed during hysteroscopic adhesiolysis. Endometrial biopsy specimens were obtained intraoperatively for histological and immunohistochemical (IHC) analysis. Clinical, hormonal, ultrasound, and Doppler parameters were assessed at baseline and at 3 and 6 months after treatment.

The primary endpoint was live birth. Secondary endpoints included clinical pregnancy rate, changes in endometrial thickness, uterine blood flow parameters (resistance index, RI), and serum anti-Müllerian hormone (AMH) levels. Additionally, potential factors associated with live birth were evaluated using regression modelling, including duration of infertility or prior pregnancy loss, severity of intrauterine adhesions, Doppler indices, and ovarian reserve markers.

In this study, the 12-month follow-up period referred to the time window during which conception after MSC therapy was monitored. All pregnancies achieved within this period were followed until their final outcome, including live birth, even if delivery occurred after the initial 12-month window. No patients were lost to follow-up during the study period.

The study was not prospectively registered in a public clinical trial registry, as it was designed as a single-arm, uncontrolled observational study without a control or randomised comparison arm. However, the study protocol, objectives, and endpoints were predefined before patient enrolment, and all data were collected prospectively.

### 2.2. Study Population

Thirty-five women of reproductive age with recurrent IUA and refractory thin endometrium were prospectively enrolled in the study. Eligible patients were aged 18–40 years and had an endometrial thickness of less than 7 mm during the implantation window (defined as 7–9 days after ovulation), the presence of intrauterine adhesions, a history of infertility and/or recurrent pregnancy loss, and at least one previous hysteroscopic adhesiolysis followed by hormonal therapy without sufficient endometrial recovery. Only women with normogonadotropic ovarian function, defined by serum gonadotropin and estradiol levels within the reference range, and without hormonal treatment within three months before enrolment were included.

Exclusion criteria were diminished ovarian reserve (anti-Müllerian hormone [AMH] <1.1 ng/mL), stage III–IV endometriosis, uterine fibroids distorting the uterine cavity, a history of malignancy, severe systemic disease, immunodeficiency, active infection (human immunodeficiency virus, hepatitis B or C, or syphilis), and male-factor infertility.

### 2.3. MSC Preparation

Human placenta samples were obtained from healthy donors aged 22–26 years (*n* = 26) following elective caesarean delivery of full-term infants at the V.I. Kulakov National Medical Research Centre for Obstetrics, Gynecology, and Perinatology. Written informed consent was obtained from all donors prior to tissue collection. All donors had an uncomplicated obstetric history, with no pregnancy complications or infectious diseases, and tested negative for hepatitis B virus (HBV), human immunodeficiency virus (HIV), and syphilis.

Placental tissue was processed under sterile conditions shortly after delivery. Samples were collected from the inner region of central placental lobules (approximately 1 cm^3^) and repeatedly washed with phosphate-buffered saline (PBS; Paneco, Moscow, Russia) to remove residual blood. The tissue fragments were mechanically minced into small pieces and subjected to enzymatic digestion with collagenase type I (100 U/mL; Gibco, Waltham, MA, USA) in serum-free Dulbecco’s Modified Eagle Medium (DMEM; Paneco, Moscow, Russia) to obtain a single-cell suspension.

After enzymatic digestion, the cell suspension was centrifuged at 300× *g* for 5 min, washed with DMEM, and centrifuged again under the same conditions. The resulting cell pellet was resuspended in DMEM/F12 medium (1:1; Paneco, Moscow, Russia) supplemented with 10% fetal bovine serum (FBS; Biosera, Cholet, France), penicillin (100 IU/mL), streptomycin (100 μg/mL) (Gibco, Waltham, MA, USA), and 2 mM L-glutamine (Paneco, Moscow, Russia).

Cells were seeded into 75 cm^2^ tissue culture flasks (Gibco Life Technologies, Waltham, MA, USA) and maintained at 37 °C in a humidified incubator with 5% CO_2_. The culture medium was replaced every 3–4 days to remove non-adherent cells and maintain optimal growth conditions. Cell morphology and proliferation were monitored regularly using phase-contrast microscopy. When cells reached approximately 80–90% confluence, they were passaged using standard enzymatic detachment procedures.

For clinical application, mesenchymal stromal cells expanded between passages 3 and 5 were used. This passage range was chosen to balance sufficient in vitro expansion with preservation of biological functionality, as early-passage MSCs retain higher proliferative capacity, stable immunophenotype, and paracrine activity, while minimising the risk of replicative senescence and phenotypic drift associated with later passages.

The immunophenotype of cultured cells met the criteria for mesenchymal stromal cells defined by the International Society for Cellular Therapy (ISCT): the cells expressed CD73, CD90, and CD105, and lacked expression of CD34, CD45, and HLA-DR, as determined by flow cytometry using a commercially available MSC Phenotyping Cocktail Kit (Miltenyi Biotec, Bergisch, Germany; REAfinity™ antibodies), according to the manufacturer’s instructions.

Safety and quality control of the cellular product were ensured through a predefined testing framework prior to clinical use. Cell viability was assessed by trypan blue exclusion using an automated cell counter (Countess II, Thermo Fisher Scientific, Waltham, MA, USA), and only preparations with viability ≥92% were released for clinical application.

Sterility testing was performed using fluid thioglycollate medium and soybean–casein digest medium in accordance with standard microbiological procedures. Screening for mycoplasma contamination was carried out using a PCR-based assay (Evrogen, Moscow, Russia).

Endotoxin levels were assessed using the limulus amebocyte lysate (LAL) assay (chromogenic method). All cell preparations met established safety criteria for parenteral administration, with endotoxin levels ≤ 5 EU/kg body weight, in accordance with accepted pharmacopoeial thresholds.

Prior to administration, each cell preparation was required to meet predefined release criteria, including viability, sterility, absence of mycoplasma contamination, and compliance with endotoxin safety limits.

### 2.4. Treatment Procedure

All patients underwent hysteroscopic evaluation during the proliferative phase of the menstrual cycle (days 7–11). When present, intrauterine adhesions were carefully dissected under direct visualization to restore the architecture of the uterine cavity.

After restoration of the uterine cavity, placenta-derived MSCs were administered using a combined local and systemic approach. A suspension containing 1 × 10^7^ MSCs in 1.7 mL of sterile physiological saline (0.9% NaCl) was injected into the endometrial–myometrial interface under hysteroscopic guidance. The cell suspension was distributed across 4–8 subendometrial injection sites along the uterine walls. Correct placement was confirmed hysteroscopically by the formation of localised subendometrial elevations. The dosing regimen was selected based on translational considerations, preclinical experience with MSC-based regenerative therapies, and the technical feasibility of multi-site subendometrial delivery under hysteroscopic guidance [26,27,28]. A fixed local dose of 1 × 10^7^ cells was chosen to ensure adequate tissue distribution while maintaining safe and technically feasible injection volumes within the subendometrial compartment.

For systemic administration, the cell dose was normalized to body weight and set at 1 × 10^6^ cells/kg, consistent with doses previously used in clinical studies of MSC-based therapies [29,30]. The calculated cell dose was administered on the following day by slow intravenous infusion in 100 mL of sterile physiological saline supplemented with 1% human serum albumin.

The combined route of administration was chosen to provide complementary local and systemic regenerative effects. Subendometrial delivery aimed to achieve direct delivery of MSCs to the injured endometrial–myometrial interface, thereby enhancing local cell retention and paracrine activity within the injured regenerative microenvironment. Intravenous administration was also used to provide systemic immunomodulatory and anti-inflammatory support, consistent with the recognised paracrine and immune-regulatory mechanisms of MSC-based therapies [31]. The dual-route strategy was considered particularly relevant for patients with severe or recurrent intrauterine adhesions, where both localised tissue remodelling and broader systemic regenerative support may be beneficial. This regimen should be regarded as exploratory, as no formal dose-ranging or route-comparison analysis was performed in this study.

Patients were closely monitored for early adverse reactions for at least 3 h after systemic administration, including assessment of vital signs and potential infusion-related reactions.

### 2.5. Ultrasonographic and Doppler Assessment

Transvaginal ultrasound examinations (frequency 5–9, 7.5 MHz, Toshiba SSA-240, Kawasaki, Japan) were performed during the implantation window (7–9 days after ovulation). Endometrial thickness was measured in the mid-sagittal plane as the maximum distance between the echogenic interfaces of the endometrium.

Colour and pulsed-wave Doppler imaging were used to assess uterine blood flow. Resistance index (RI) values were obtained from the uterine, arcuate, and radial arteries. Measurements were obtained at baseline, and at 3 and 6 months following therapy.

### 2.6. Histological and Immunohistochemical Analysis

Endometrial biopsies were obtained during hysteroscopy prior to MSC administration. Tissue samples were fixed in 10% neutral buffered formalin, embedded in paraffin, and sectioned at a thickness of 4–5 μm.

Routine haematoxylin and eosin staining was performed for general morphological assessment. Stromal fibrosis was evaluated using collagen-specific staining methods (Heidenhain’s azan and Weigert–Van Gieson). Quantitative morphometric analysis was conducted to assess the percentage and absolute area of collagen deposition within the endometrial stroma. Measurements were assessed using digital image analysis, and collagen content was expressed both as the percentage of stained area and as total collagen area (mm^2^).

IHC analysis was performed to detect MUM1-positive plasma cells as a marker of chronic endometritis. Nuclear immunoreactivity was considered positive. The number of positive cells was quantified per 10 high-power fields (×400 magnification). The presence of ≥5 positive plasma cells per 10 high-power fields was interpreted as consistent with chronic endometritis.

Histological, immunohistochemical, and morphometric assessments were performed by experienced pathologists blinded to clinical and reproductive outcomes to minimise potential assessment bias.

### 2.7. Hormonal Assessment

AMH levels were measured to assess ovarian reserve and to evaluate their potential association between AMH levels and reproductive outcomes following MSC therapy. Venous blood samples were obtained at baseline (prior to treatment) and at 3 and 6 months after MSC administration. As AMH levels are relatively cycle-independent, sampling was performed irrespective of the menstrual cycle phase. AMH concentrations were determined using a commercially available enzyme-linked immunosorbent assay according to the manufacturer’s instructions. All analyses were conducted in the same laboratory to ensure consistency.

### 2.8. Outcomes

**Figure 1:**
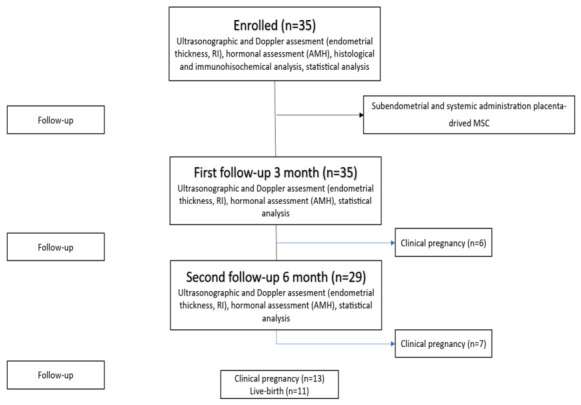
Flow diagram of patient enrolment, treatment, and reproductive outcomes after placenta-derived mesenchymal stromal cell therapy.

The primary endpoint of the study was live birth, defined as the delivery of a live-born infant after 24 weeks of gestation. Secondary endpoints included clinical pregnancy rate and time to pregnancy following MSC therapy. Univariable logistic regression analyses were performed to explore associations between candidate baseline factors and live birth. Menstrual function and recurrence of intrauterine adhesions were analyzed descriptively. The overall study design is presented in Figure 1.

Thirty-five women with intrauterine adhesions and thin endometrium were enrolled and underwent baseline ultrasonographic and Doppler evaluation (endometrial thickness and uterine, arcuate, and radial arteries RI), hormonal assessment (anti-Müllerian hormone), and histological and immunohistochemical analyses. All patients received combined subendometrial and systemic administration of placenta-derived MSCs. Follow-up assessments were performed at 3 months (*n* = 35) and 6 months (*n* = 29). During the observation period, 13 clinical pregnancies were achieved, resulting in 11 live births.

### 2.9. Statistical Analysis

Statistical analyses were performed using R software (version 4.5.2; R Foundation for Statistical Computing, Vienna, Austria). Continuous variables are presented as median (interquartile range, IQR), and categorical variables as counts and percentages. Between-group comparisons were performed using the Mann–Whitney U test for continuous variables and Fisher’s exact test for categorical variables.

Within-subject changes in endometrial thickness from baseline to 3 and 6 months after MSC administration were assessed using the Wilcoxon signed-rank test for paired data. Absolute changes in endometrial thickness were calculated as the difference between follow-up and baseline values.

The primary outcome was live birth. Associations between candidate baseline factors and live birth were assessed using univariable logistic regression models, reporting odds ratios (ORs) with 95% confidence intervals (CIs). Skewed variables were log_2_-transformed where appropriate. For the AFS score, Firth’s logistic regression method was applied due to the limited number of outcome events and complete separation of outcomes between groups.

Given the small number of live birth outcomes (11 cases), the number of variables included in regression modelling was restricted to minimise the risk of overfitting. Because only univariable models were applied, each model included a single factor, resulting in an events-per-variable (EPV) ratio of 11. In contrast, inclusion of multiple factors in a multivariable model would have substantially reduced the EPV ratio and increased the risk of overfitting and unstable estimates; therefore, multivariable regression was not performed. Consequently, adjustment for potential confounders was not possible, and all regression findings were interpreted as exploratory associations rather than independent prognostic effects or causal effects.

For categorical predictors with sparse cells or complete separation, sensitivity analyses were performed using the Haldane–Anscombe continuity correction by adding 0.5 to each cell of the 2 × 2 table. Fisher’s exact test was additionally used to assess the robustness of associations involving sparse categorical variables.

Time to live birth was analysed using Kaplan–Meier methods. A two-sided *p*-value < 0.05 was considered statistically significant.

## 3. Results

### 3.1. Baseline Characteristics

The study included 35 women with recurrent IUA and refractory thin endometrium. The median age of the participants was 36 years (IQR 32–40).

Menstrual dysfunction was predominantly characterized by hypomenorrhoea, observed in 22 patients (62.9%), while secondary amenorrhoea was diagnosed in 13 patients (37.1%). Reproductive disorders were present in all participants. Isolated infertility was documented in 12 patients (34.3%), and a history of one or more clinical pregnancy losses was recorded in 6 patients (17.1%). A combination of infertility and prior pregnancy loss was observed in 17 patients (48.6%). Additionally, eight patients (22.8%) had a history of unsuccessful assisted reproductive technology (ART) attempts. The median duration of infertility and/or previous pregnancy loss was 3 years (IQR 1–5).

Hysteroscopic evaluation showed that most patients had recurrent intrauterine adhesions of mild severity. According to the American Fertility Society (AFS) classification, adhesions were grade I in 26 patients (74.3%) and grade II in 9 patients (25.7%).

Baseline ultrasonographic and Doppler findings indicated marked impairment of endometrial receptivity and uterine perfusion in the study population. The median endometrial thickness during the implantation window was 4 mm (IQR 2.35–6). Baseline RI values in the uterine arteries and the myometrial–subendometrial arterial network were within the range characteristic of impaired endometrial perfusion. The median anti-Müllerian hormone level was 1.44 ng/mL (IQR 1.1–2.36), indicating preserved ovarian reserve in most participants.

Histological examination most frequently revealed fibrous–sclerotic remodeling of the endometrial stroma, characterized by focal fibrosis and lymphoid infiltration. Histochemical staining demonstrated collagen deposition in 48.1% of the examined samples. Immunohistochemical analysis showed signs of chronic endometritis, identified by the presence of MUM1-positive plasma cells, in 23.5% of patients. However, due to the limited number of samples for MUM1 assessment, the results should be interpreted with caution.

Detailed results of the quantitative morphometric and immunohistochemical analyses are presented in Supplementary Table S1. Notably, no statistically significant association was found between quantitative fibrosis parameters and reproductive outcomes in this cohort.

### 3.2. Reproductive Outcomes

The primary clinical outcome of the study was live birth. During the 12-month observation period, clinical pregnancy occurred in 13 of 35 patients (37.1%; 95% CI 21.5–55.1). Clinical pregnancy was defined as ultrasound confirmation of a gestational sac. Live birth was achieved in 11 patients (31.4%; 95% CI 16.9–49.3).

Of the successful pregnancies, eight deliveries were vaginal and three were by caesarean section. All caesarean sections were planned procedures. All births occurred after 37 weeks of gestation. No cases of preterm birth, pre-eclampsia, severe placental insufficiency, or other serious obstetric complications were recorded. The neonatal period was uneventful, with no cases of congenital anomalies or need for neonatal intensive care.

Two pregnancies resulted in early pregnancy loss. One patient experienced spontaneous miscarriage at 5–6 weeks of gestation, while another had a missed abortion at 6–7 weeks of gestation. The first patient had a history of secondary amenorrhoea, recurrent pregnancy loss, and infertility, whereas the second patient had hypomenorrhoea and infertility.

### 3.3. Changes in Endometrial Thickness After MSC Administration

Endometrial thickness was analysed as a predefined secondary outcome. Median endometrial thickness increased from 4.0 mm (IQR 2.35–6.0) at baseline to 6.2 mm (IQR 5.1–7.0) at 3 months and 6.6 mm (IQR 5.0–7.0) at 6 months after MSC administration. The increase was statistically significant both from baseline to 3 months and from baseline to 6 months (*p* < 0.001 for both comparisons).

The median absolute increase in endometrial thickness at 3 months was 2.0 mm (IQR 0.75–3.25) in the overall cohort. When stratified by live birth status, the increase was 1.2 mm (IQR 0.2–2.15) among patients who did not achieve live birth and 3.5 mm (IQR 1.7–4.0) among patients who achieved live birth. The magnitude of increase was significantly greater in the live birth group (*p* = 0.007). Within-group changes from baseline were statistically significant both in patients without live birth (*p* < 0.001) and in those with live birth (*p* = 0.002).

In exploratory univariable logistic regression analysis, baseline endometrial thickness was not significantly associated with live birth (OR = 0.81; *p* = 0.305). These findings indicate that although endometrial thickness increased during follow-up after MSC administration, baseline endometrial thickness alone was not associated with the probability of live birth in this cohort. Because the study lacked a control group, the observed increase in endometrial thickness should be interpreted as a within-subject change after MSC administration rather than as evidence of a causal treatment effect.

### 3.4. Baseline Characteristics According to Live Birth

**Figure 2:**
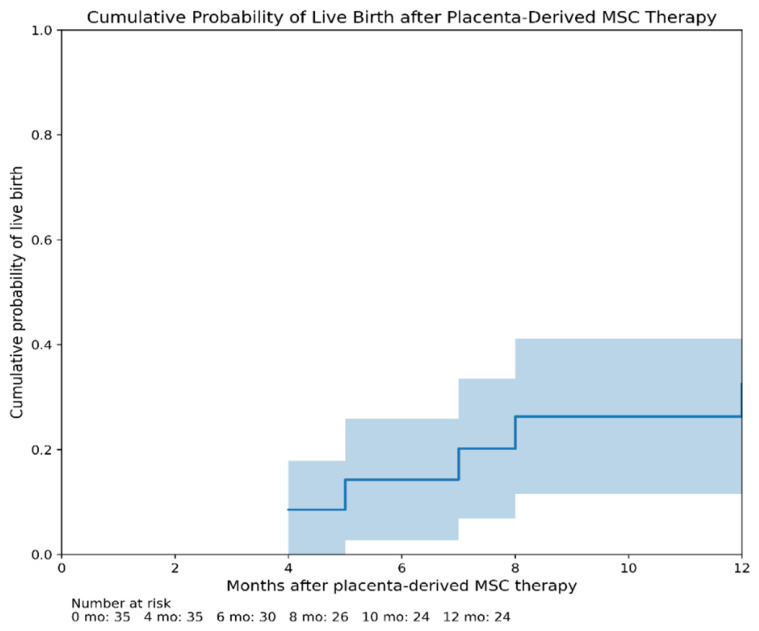
Cumulative probability of live birth after placenta-derived mesenchymal stromal cell therapy. Kaplan–Meier analysis of time to live birth in 35 patients treated with placenta-derived mesenchymal stromal cells. The shaded areas indicate 95% confidence intervals. Patients who did not achieve live birth during the 12-month follow-up were censored at the end of observation. The number of patients at risk at selected time points is shown below the plot.

Clinical pregnancies occurred 4–12 months after initiation of MSC therapy. The median interval between cell therapy and clinical pregnancy was 7 months (IQR 5–8). The most pregnancies occurred relatively early after treatment, indicating that the likelihood of missing late deliveries is low and that the 12-month observation window was sufficient to capture clinically relevant reproductive outcomes. Four pregnancies (30.8%) occurred after transfer of cryopreserved embryos, while the remaining nine pregnancies (69.2%) occurred spontaneously within 6–12 months after MSC therapy (Figure 2).

To identify factors associated with live birth, patients were stratified according to reproductive outcome (live birth vs. no live birth). A comparative analysis of baseline clinical and functional parameters between the two groups is presented in Table 1.

Patients who achieved live birth had a significantly shorter duration of reproductive disorders (*p* = 0.044) and a higher prevalence of stage I intrauterine adhesions according to the AFS classification (*p* = 0.033). In addition, this subgroup demonstrated lower baseline RI values in the uterine, arcuate and radial arteries, as well as higher serum AMH levels.

### 3.5. Baseline Factors Associated with Live Birth

To explore factors associated with the probability of live birth after placenta-derived MSC administration, univariable logistic regression analyses were performed. The analysis included baseline clinical, hysteroscopic, Doppler, hormonal, histological, and immunohistochemical parameters potentially related to reproductive outcome. Because only univariable models were used, these analyses did not allow adjustment for potential confounders. Therefore, the observed associations should be interpreted as exploratory and hypothesis-generating rather than as independent predictors of live birth or evidence of a causal treatment effect.

**Figure 3:**
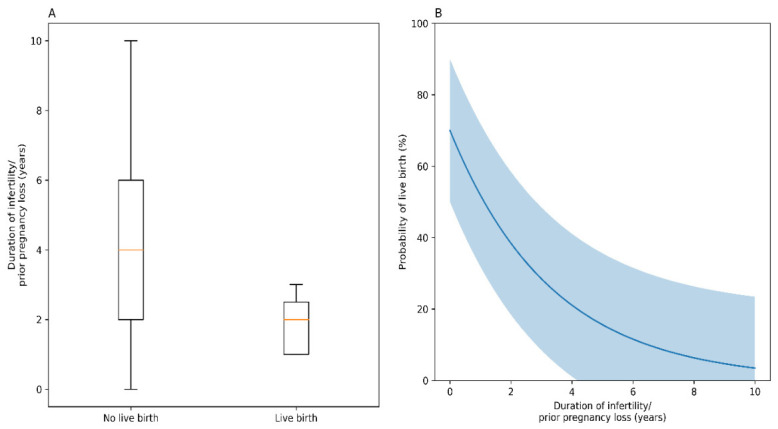
Association between duration of infertility and/or prior pregnancy loss and the probability of live birth. (**A**) Distribution of infertility and/or prior pregnancy loss duration in patients who achieved live birth and those who did not. (**B**) Predicted probability of live birth according to the duration of infertility and/or prior pregnancy loss, based on univariable logistic regression analysis. The shaded area indicates the 95% confidence interval.

Patients who achieved live birth had a shorter duration of infertility and/or prior pregnancy loss than those who did not achieve live birth (2 [1–3] years vs. 4 [1.25–6.5] years, *p* = 0.044). The median difference between the groups was 2 years (95% CI: −1 to 4). Each additional year of reproductive disorder duration was associated with higher odds of not achieving live birth (OR 1.55; 95% CI: 1.10–2.57; *p* = 0.038) (Figure 3). Conversely, a shorter duration of reproductive disorders before MSC administration was associated with live birth in this exploratory analysis.

Hysteroscopic, histological, and IHC parameters were evaluated as potential factors associated with treatment success evaluated as potential associations with treatment success. All live births occurred in patients with AFS stage I adhesions, whereas no live births were observed among patients with AFS stage II adhesions, resulting in complete separation in the conventional logistic regression model. To address this issue, Firth’s penalized logistic regression was applied. In this analysis, AFS stage I was associated with higher odds of live birth compared with AFS stage II adhesions (OR 6.8; 95% CI 1.1–42; *p* = 0.04).

As a sensitivity analysis, the association was additionally assessed using the Haldane–Anscombe continuity correction. This analysis yielded a similar direction of association, with AFS stage I associated with higher odds of live birth compared with stage II adhesions (OR 14.1; 95% CI 0.74–267.8). Fisher’s exact test yielded *p* = 0.033. However, the wide confidence intervals reflect the small sample size and the absence of live birth events in the AFS stage II subgroup; therefore, this finding should be interpreted as exploratory and requiring confirmation in larger controlled studies. In contrast, no statistically significant association was found between quantitative fibrosis parameters and reproductive outcome. In contrast, no statistically significant association was found between quantitative fibrosis parameters and reproductive outcome.

Baseline uterine perfusion parameters showed a consistent association with live birth. Lower baseline RI values in the right and left uterine arteries, as well as in the arcuate and radial arteries, were significantly associated with an increased probability of live birth. Specifically, each increase in RI by 0.01 was associated with reduced odds of live birth: 1.26-fold for the right uterine artery (95% CI: 1.07–1.56; *p* = 0.012), 1.31-fold for the left uterine artery (95% CI: 1.11–1.64; *p* = 0.006), 1.26-fold for the arcuate arteries (95% CI: 1.09–1.53; *p* = 0.006), and 1.22-fold for the radial arteries (95% CI: 1.06–1.48; *p* = 0.019). Overall, these findings indicate that better baseline uterine and subendometrial blood flow was associated with a greater likelihood of live birth.

**Figure 4:**
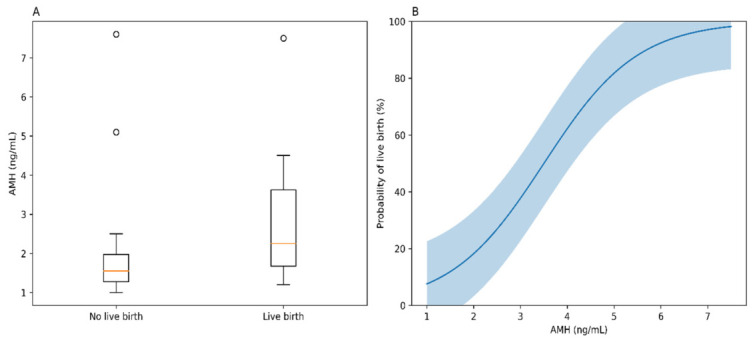
Association between baseline serum anti-Müllerian hormone levels and the probability of live birth. (**A**) Distribution of baseline serum AMH levels in patients who achieved live birth and those who did not. (**B**) Predicted probability of live birth according to baseline AMH level, based on univariable logistic regression analysis. The shaded area indicates the 95% confidence interval.

Baseline AMH levels also demonstrated a statistically significant association with live birth. A twofold increase in AMH concentration was associated with a 2.59-fold increase in the odds of live birth (*p* = 0.032) (Figure 4). This suggests that a more favourable ovarian reserve profile at baseline may be associated with improved reproductive outcomes after therapy.

Thus, the clinical and functional factors associated with live birth following MSC therapy included a shorter duration of infertility or prior pregnancy loss, stage I intrauterine adhesions according to the AFS classification, lower baseline RI values in the uterine, arcuate, and radial arteries, and higher baseline AMH levels.

**Figure 5:**
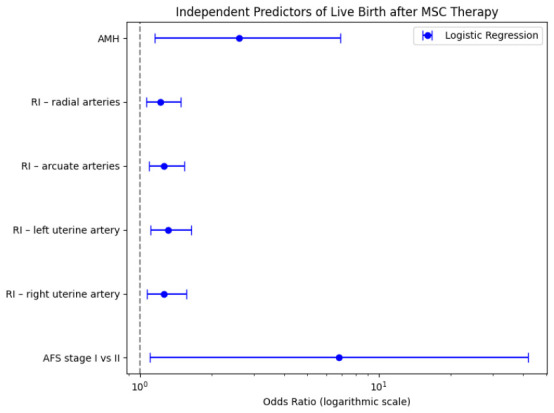
Forest plot of factors associated with live birth after placenta-derived mesenchymal stromal cell therapy. Odds ratios (points) and 95% confidence intervals (horizontal lines) are shown from univariable logistic regression analysis for baseline associations with live birth, with Firth’s regularization applied for the AFS variable. The vertical dashed line indicates an odds ratio of 1 (no association). RI values represent the resistance index in the corresponding uterine, arcuate, and radial arteries; AMH is modeled on a log_2_ scale (per doubling).

The results of the univariable logistic regression analyses are summarised in Table 2. The magnitude and direction of the associations between clinical and functional parameters and the probability of live birth are illustrated in Figure 5.

### 3.6. Safety and Tolerability

Placenta-derived MSC therapy was generally well tolerated. No treatment-related serious adverse events, including anaphylactic reactions, thromboembolic events, or infectious complications, were observed during the treatment period or follow-up. Subendometrial administration under hysteroscopic guidance was not associated with uterine perforation, excessive bleeding, or postoperative pelvic inflammatory complications, and systemic intravenous infusion was also well tolerated. Transient low-grade fever (≤37.8 °C) occurred in five patients (14.3%) within 1–2 h after systemic MSC administration; in all cases, body temperature normalised spontaneously within several hours without pharmacological intervention. Similar reactions were not observed after surgery and subendometrial administration of MSCs, suggesting that this reaction was likely related to the systemic administration of the cells.

No hospital readmissions or treatment discontinuations due to adverse events were recorded. During follow-up, no cases of abnormal tissue proliferation, endometrial hyperplasia, or suspected malignant transformation were detected. Overall, the combined local and systemic administration of placenta-derived MSCs demonstrated a favourable safety and tolerability profile in this cohort.

## 4. Discussion

This single-arm uncontrolled observational study demonstrated that therapy with placenta-derived MSCs in patients with recurrent IUA was associated with a live birth rate of 31.4%. Notably, the study cohort consisted exclusively of patients with poor reproductive prognosis and marked therapeutic resistance. All participants had previously undergone one to five surgical adhesiolysis procedures, as well as hormonal and, in some cases, adjunctive therapies, without achieving a sustained clinical effect.

The characteristics of the study population, representing a cohort with an unfavorable clinical prognosis, significantly impact the interpretation of the results. All patients had recurrent intrauterine adhesions, thin endometrium (<7 mm), and a history of at least one, and up to five, failed hysteroscopic adhesiolysis procedures. Notably, a significant proportion of the patients (8/13) who achieved pregnancy in our cohort had undergone multiple failed surgical interventions (3 had 2, 2 had 3, and 3 had 3 surgical adhesiolyses).

The observed live birth rate of 31.4% falls within the range reported in recent systematic reviews and meta-analyses evaluating MSC therapy for intrauterine adhesions and thin endometrium [19,21,22]. In the systematic review and meta-analysis by Yuan et al., the cumulative live birth rate reached 40% (95% CI 22–60) and 37% (95% CI 0.25–0.50) in the subgroup of patients with recurrent adhesions [21]. However, direct comparison with the results reported by Yuan et al. is limited by differences in study design, particularly the absence of a control group in the present study [21]. Similarly, a meta-analysis by Adamyan et al. demonstrated a significant increase in the odds of live birth compared with standard treatment (OR = 2.27; *p* = 0.01) together with an increase in endometrial thickness [22]. Comparable trends were reported by Gao et al., although these high relative risk estimates should be interpreted cautiously due to the limited number of controlled studies and the potentially low baseline live birth rates in control groups [19].

Available literature shows that reproductive outcomes after hysteroscopic adhesiolysis are highly dependent on the severity of the condition and the frequency of recurrences. In a large prospective cohort study, the overall conception rate after adhesiolysis was 48.2%, but it significantly decreased with increasing severity of adhesions, reaching only 25% in patients with severe intrauterine adhesions. Furthermore, 7.9% of patients experienced obstetric complications, including postpartum hemorrhage due to placenta accreta or placental adhesions [4]. Similarly, Roy et al. reported a conception rate of 40.4%, with significantly lower rates in moderate and severe cases (approximately 30–33%) [3]. Notably, the need for repeated adhesiolysis procedures was consistently associated with unfavorable prognosis. In Roy et al.’s study, no pregnancies were recorded in patients requiring repeat adhesiolysis, highlighting the negative impact of adhesion recurrence and the necessity of multiple surgical interventions on fertility outcomes. Additionally, in this study, four (12.5%) patients experienced postpartum hemorrhage due to placental accreta [3]. Moreover, thin endometrium is a well-established independent factor associated with low implantation and pregnancy rates, further exacerbating the poor prognosis in this patient group.

In our study, no complications were observed in the postpartum period for patients whose pregnancies resulted in live birth. All deliveries were full-term, with no cases of preterm birth, preeclampsia, or serious obstetric complications such as placental insufficiency.

In summary, these data suggest that patients with recurrent intrauterine adhesions, thin endometrium, and previous failed adhesiolysis procedures represent a subgroup with a significantly lower baseline probability of spontaneous conception compared to the general post-adhesiolysis population. In this context, the observed pregnancy rate (37.1%) and live birth rate (31.4%) in our study should be considered exploratory associations, suggesting the potential of cell therapy to improve reproductive outcomes in this patient group and opening opportunities for further studies to confirm its efficacy in controlled trials.

The combined subendometrial and intravenous administration employed in this study should be considered an exploratory therapeutic strategy rather than an optimized delivery protocol. The subendometrial route was selected to place MSCs in close proximity to the injured endometrium, where greater local retention and paracrine support of angiogenesis, stromal remodeling, and tissue repair may promote regeneration [32,33,34]. The intravenous route was intended to provide broader immunomodulatory and anti-inflammatory effects, consistent with the view that MSCs may exert part of their therapeutic benefit through transient endocrine and paracrine signaling even when durable engraftment is limited [33,35]. At the same time, intravenously administered MSCs are known to undergo substantial pulmonary first-pass sequestration, and the extent to which the systemically delivered cell dose in our study reached uterine tissue remains uncertain [33,36,37,38]. In addition, although preclinical studies in thin endometrium have suggested potential benefits of systemic MSC administration [39], these findings do not in themselves demonstrate efficient uterine homing. Because biodistribution and cell-tracking analyses were not performed, and no comparator groups were included to assess route-specific effects, the relative contributions of local and systemic administration, as well as the optimal delivery route, cannot be determined from the present study.

Identification of baseline factors associated with reproductive outcomes after MSC administration is clinically important for future patient stratification and study design. In the present study, favorable outcomes (live birth) were associated with AFS grade I IUA, shorter duration of reproductive disorders, lower baseline RI values in uterine, arcuate and radial arteries, and higher AMH levels. However, these associations were derived from univariable models and therefore could not be adjusted for potential confounding between clinical, vascular, hormonal, and hysteroscopic factors. Consequently, they should be interpreted as exploratory and hypothesis-generating, rather than as independent prognostic factors or evidence of treatment efficacy.

These results support a stratified approach to placenta-derived MSC therapy. Patients with recurrent IUA and refractory thin endometrium represent a heterogeneous population with varying degrees of structural and functional endometrial damage. Our findings suggest that regenerative therapy may be associated with better outcomes when microcirculation is relatively preserved, the adhesive process is limited, and ovarian reserve remains sufficient.

Although quantitative measures of collagen deposition assessed using Heidenhain and Weigert–Van Gieson staining were not significantly associated with live birth in this study, the significant association with reproductive outcome was observed for the severity of intrauterine adhesions according to the AFS classification (*p* = 0.04). Notably, no live births occurred among patients with grade II IUA. The association between AFS stage and live birth should therefore be interpreted with particular caution. No live births occurred among patients with AFS stage II adhesions, resulting in complete separation. Although the sensitivity analysis using a continuity-corrected estimate and Fisher’s exact test was consistent with the observed association, the confidence interval was very wide, indicating substantial statistical uncertainty. Therefore, AFS stage should be considered an exploratory baseline factor related to live birth in this cohort, and this finding requires confirmation in larger controlled studies. This finding suggests that the clinical severity of the adhesive process may reflect deeper and more extensive fibrotic remodeling of the endometrium, including damage to the basal layer and vascular network. Such changes may not be fully captured by morphometric assessment of biopsy samples.

The lack of a significant association between collagen quantification and pregnancy outcomes may be explained by several factors. These include the limited number of analyzed samples, the mosaic distribution of fibrosis, and the fact that morphometry mainly reflects the area of collagen deposition rather than the spatial organisation of the stromal matrix, microvascular integrity, or the molecular profile of the tissue. In this context, the observed association between uterine blood flow parameters and reproductive outcomes suggests that functional characteristics of the endometrium may more accurately reflect implantation potential than static measurements of fibrotic transformation.

Indeed, lower baseline RI values in the uterine, arcuate, and radial arteries were associated with a higher probability of live birth. Conversely, an increase in RI by 0.01 was associated with a 1.2–1.3-fold decrease in the odds of a favourable outcome. These findings are consistent with those reported by Smart et al., who demonstrated higher uterine artery RI and pulsatility index (PI) values in women with unexplained infertility compared with fertile controls [40]. Increased vascular resistance was associated with impaired endometrial perfusion and reduced implantation potential. It is important to note that the resistance index (RI) may partially reflect the severity of intrauterine adhesions and the underlying structural damage, rather than being a purely independent factor. Therefore, uterine blood flow parameters should be considered not only as diagnostic indicators but also as potential prognostic markers of reproductive outcome, reflecting the functional readiness of the endometrium to respond to regenerative therapy.

Another important observation was the significant association between the duration of reproductive disorders and the probability of live birth following placenta-derived MSC therapy. A longer duration of infertility and/or recurrent pregnancy loss was associated with a lower likelihood of live birth (OR 1.55 for each additional year; *p* = 0.038). This finding suggests that the temporal factor reflects not only clinical history but also the degree of accumulated morphofunctional damage in the endometrium. Prolonged reproductive disorders may therefore represent the cumulative effects of chronic injury to the basal endometrial layer and progressive depletion of its regenerative potential.

From a pathophysiological perspective, MSC therapy may be most effective when endometrial damage remains partially reversible and residual regenerative and angiogenic capacity is preserved. As the disease progresses and structural sclerosis becomes more pronounced, the therapeutic effect of MSCs may decrease due to extensive extracellular matrix remodeling and impaired vascular reactivity [41]. Thus, both the duration of disease and the clinical severity of adhesions may reflect the degree of irreversible endometrial damage.

Although patients with diminished ovarian reserve were excluded from the study, higher baseline AMH levels were significantly associated with the probability of live birth after MSC therapy. A twofold increase in AMH concentration was associated with more than a twofold increase in the odds of live birth. These findings suggest that ovarian reserve may influence not only overall reproductive potential but also the response to regenerative endometrial therapy.

AMH reflects the functional pool of preantral and small antral follicles and indirectly characterises the endocrine environment in which endometrial regeneration occurs. Higher ovarian reserve is associated with more stable steroid support and likely with more favourable angiogenic and immunoregulatory conditions. These factors may enhance the paracrine effects of MSCs. This interpretation is consistent with studies investigating MSC therapy in patients with diminished ovarian reserve and premature ovarian insufficiency, where improvements in hormonal parameters and ovarian function have been reported after cell therapy [42]. Overall, these findings highlight the importance of considering MSC therapy within the broader context of interactions between local regenerative processes and systemic reproductive status.

The selection of placenta-derived MSCs as the cellular source is justified both biologically and technologically. Perinatal tissues demonstrate high proliferative capacity, a strong paracrine secretory profile, and low immunogenicity. They are suitable for prolonged in vitro expansion and exhibit pronounced immunomodulatory properties [24,43,44], making them promising candidates for allogeneic therapeutic protocols. Experimental studies have shown that placenta-derived MSCs activate signaling pathways involved in angiogenesis and cell proliferation, including the JNK/ERK1/2–STAT3–VEGF and JAK2–STAT5 pathways, thereby increasing VEGF expression and stimulating neoangiogenesis [35]. These mechanisms are pathogenetically relevant for conditions characterized by impaired endometrial perfusion and fibrotic remodeling.

The combined local and systemic administration strategy was based on biological plausibility rather than on a previously established optimized protocol. Local subendometrial injection was intended to increase MSC exposure at the damaged endometrial–myometrial interface, whereas systemic infusion was included to provide potential trophic, pro-angiogenic, and immunomodulatory support. The local dose of 1 × 10^7^ cells was selected as a clinically feasible fixed dose for multi-site hysteroscopically guided injection and was informed by previous studies using local MSC-based delivery for endometrial repair. The systemic dose of 1 × 10^6^ cells/kg was chosen because weight-adjusted doses in this range are commonly used in clinical MSC administration protocols. However, this dual-route regimen should be regarded as exploratory rather than optimized, and the present study was not designed to determine the independent contribution of local and systemic administration to the observed reproductive outcomes.

The favourable safety profile observed in this study further supports the clinical applicability of placenta-derived MSC therapy. No serious adverse events were recorded. Only transient subfebrile reactions occurred in several patients following systemic MSC administration, consistent with previously reported findings [45].

From a technological perspective, the placenta represents a highly scalable source of MSCs. Experimental studies indicate that a single full-term placenta can yield more than 7000 clinical doses of MSCs using automated isolation systems and bioreactor-based expansion [24]. This scalability is essential for the development of standardized allogeneic cell therapy products.

According to the current literature, clinical studies specifically evaluating placenta-derived MSCs for the treatment of intrauterine adhesions and refractory thin endometrium remain scarce. Most clinical studies in this field have investigated MSCs derived from bone marrow, umbilical cord, menstrual blood, or endometrial tissue [19,21,22,46]. The present study therefore expands the current evidence base by providing clinical data on the use of placenta-derived MSCs in patients with severe endometrial insufficiency and unfavourable reproductive history. The use of live birth as the primary clinical outcome further strengthens the clinical relevance of these findings.

### Strengths and Limitations

The strengths of this study include its prospective design, the evaluation of live birth as the primary clinical outcome, and the exploratory assessment of vascular parameters potentially associated with reproductive outcomes after MSC administration. However, several limitations should be considered when interpreting the results.

First, the sample size was relatively small, and the limited number of live birth events constrained the statistical analysis. This reduced statistical power and resulted in wide confidence intervals for several estimated associations. To minimise the risk of overfitting, only univariable logistic regression models were applied, with one variable included per model, resulting in an events-per-variable ratio of 11 for each analysis. Multivariable regression was not performed because it would have substantially increased the risk of model instability and overfitting. Consequently, adjustment for potential confounders was not possible, and the identified associations should be interpreted as exploratory rather than as independent prognostic factors or causal effects.

This limitation was particularly relevant for the analysis of AFS stage, as no live births occurred in the AFS stage II subgroup, resulting in complete separation. Although a sensitivity analysis using the Haldane–Anscombe continuity correction and Fisher’s exact test was performed to address this issue, the resulting estimate should still be interpreted cautiously.

Second, the study lacked a control group, precluding definitive conclusions regarding the causal effect of mesenchymal stromal cell administration on reproductive outcomes. Therefore, the findings should be interpreted as exploratory associations rather than conclusive evidence of treatment efficacy.

Third, all patients received the same combined local and systemic MSC regimen. Therefore, the study could not assess dose–response relationships or determine the independent contribution of subendometrial versus systemic administration to the observed reproductive outcomes. This represents an important limitation for optimisation of the dose, route, and timing of placenta-derived MSC administration.

Fourth, the study was conducted at a single centre, which may limit the generalisability of the results to broader patient populations. The eligibility criteria also restricted the study population. Patients with diminished ovarian reserve, male-factor infertility, and stage III–IV endometriosis were excluded to reduce clinical heterogeneity and limit the influence of major non-endometrial infertility factors. While this approach improved internal validity, it limits the generalisability of the findings to real-world populations with recurrent IUA and refractory thin endometrium, in whom these comorbidities may coexist. The exclusion of patients with diminished ovarian reserve is particularly relevant, as baseline AMH levels remained significantly associated with live birth even within the included cohort, underscoring the importance of systemic reproductive factors. Accordingly, the present results should be interpreted as applicable primarily to patients meeting similar eligibility criteria and should not be extrapolated to patients with these excluded comorbidities without further study.

Finally, the study population included patients with heterogeneous reproductive histories, including infertility and recurrent pregnancy loss, which may have increased clinical variability and influenced the observed associations. Future controlled studies including larger and more diverse populations, including patients with a broader range of ovarian reserve, are needed to assess the robustness and generalisability of these findings.

## 5. Conclusions

This prospective single-arm uncontrolled observational study found that placenta-derived MSC therapy was followed by a live birth rate of 31.4% in women with recurrent IUA and refractory thin endometrium who had an unfavorable reproductive prognosis and prior resistance to standard treatment. Because the study lacked a control group, these findings should be interpreted as exploratory and hypothesis-generating only and do not establish the causal efficacy of MSC therapy.

Favorable reproductive outcomes after MSC administration were associated with shorter duration of reproductive disorders, AFS stage I intrauterine adhesions, lower baseline uterine, arcuate, and radial artery RI values, and higher baseline AMH levels. These associations suggest that baseline endometrial perfusion, adhesion severity, and systemic reproductive status may be related to the probability of live birth after MSC administration. However, because only univariable models were used, these factors should not be interpreted as independent predictors.

The present findings should be considered applicable primarily to patients meeting similar eligibility criteria and should not be directly generalized to broader clinical populations without further study. Future multicenter controlled studies, preferably with randomized or matched comparison groups, are required to determine whether placenta-derived MSC therapy improves reproductive outcomes beyond the expected prognosis after standard treatment, to assess dose–response relationships and the relative contribution of local and systemic administration, and to clarify the mechanisms underlying the observed associations.

## Figures and Tables

**Table 1:** Baseline clinical and functional characteristics of patients according to reproductive outcome (live birth vs. no live birth). Note: Continuous variables are presented as median (interquartile range, IQR), and categorical variables as number (percentage). The Mann–Whitney U test for continuous variables and Fisher’s exact test for categorical variables were used to compare groups. *p*-values represent comparisons between the live birth and no live birth groups. *n* represents the total number of participants in each group. BMI—body mass index; AMH—anti-Müllerian hormone; RI—resistance index; AFS—American Fertility Society classification.

Variable	Total (*n* = 35)	No Live Birth (*n* = 24)	Live Birth (*n* = 11)	*p*-Value
Age (years), median (IQR)	36 (32–40)	37 (33.5–40)	34 (31.5–37.5)	0.283
BMI (kg/m^2^), median (IQR)	22 (20–25)	22 (20–25)	22 (20–22.6)	0.955
Duration of infertility/prior pregnancy loss (years), median (IQR)	3 (1–5)	4 (1.25–6.5)	2 (1–3)	0.044
AFS stage I, *n* (%)	26 (74.3%)	15 (62.5%)	11 (100%)	0.033
AFS stage II, *n* (%)	9 (25.7%)	9 (37.5%)	-	0.052
Baseline endometrial thickness (mm), median (IQR)	4 (2.35–6)	4.3 (2.2–6)	3 (2.7–5)	0.379
Baseline AMH (ng/mL), median (IQR)	1.44 (1.1–2.36)	1.15 (1.1–1.91)	1.9 (1.6–4.2)	0.008
Baseline RI—right uterine artery	0.85 (0.81–0.90)	0.86 (0.84–0.90)	0.82 (0.78–0.85)	0.004
Baseline RI—left uterine artery	0.85 (0.80–0.88)	0.87 (0.85–0.90)	0.80 (0.77–0.82)	0.001
Baseline RI—arcuate arteries	0.75 (0.70–0.80)	0.78 (0.74–0.80)	0.70 (0.67–0.70)	0.002
Baseline RI—radial arteries	0.68 (0.61–0.70)	0.69 (0.66–0.70)	0.62 (0.60–0.65)	0.011

**Table 2:** Univariable logistic regression of baseline factors associated with live birth after placenta-derived MSC-therapy with firth’s regularization for one predictor. Note: Odds ratios (OR) represent the change in odds of live birth associated with each predictor. Duration of infertility/prior pregnancy loss is expressed per additional year. RI values represent the resistance index in the corresponding uterine or subendometrial arteries. AFS, American Fertility Society classification; RI, resistance index; AMH, anti-Müllerian hormone. Primary analysis estimates shown; continuitly-corrected sensitivity estimate described in text.

Variable	OR	95% CI	*p*-Value
Duration of infertility/prior pregnancy loss (per year)	1.55	1.10–2.57	0.038
AFS stage I vs. II	6.8	1.1–42	0.04
RI—right uterine artery	1.26	1.07–1.56	0.012
RI—left uterine artery	1.31	1.11–1.64	0.006
RI—arcuate arteries	1.26	1.09–1.53	0.006
RI—radial arteries	1.22	1.06–1.48	0.019
AMH	2.59	1.15–6.89	0.032

## Data Availability

The main data supporting the findings of this study are included in the article. Additional data are available from the corresponding authors upon reasonable request.

## Supplementary Materials

The following supporting information can be downloaded at: https://www.mdpi.com/article/10.3390/life16060871/s1, Table S1. Morphological and immunohistochemical characteristics of the endometrium according to reproductive outcome.

## Abbreviations

The following abbreviations are used in this manuscript:

AFS	American Fertility Society
AMH	anti-Müllerian hormone
ART	assisted reproductive technology
BMI	body mass index
CI	confidence interval
DMEM	Dulbecco’s Modified Eagle Medium
ELISA	enzyme-linked immunosorbent assay
EPV	events-per-variable
FBS	fetal bovine serum
HBV	hepatitis B virus
HIV	human immunodeficiency virus
HPF	high-power field
IHC	immunohistochemical
IUA	intrauterine adhesions
MSC	mesenchymal stromal cell
OR	odds ratio
PBS	phosphate-buffered saline
RI	resistance index
