# Supplementary material for: Factors Associated with Live Birth After Placenta-Derived Mesenchymal Stromal Cell Therapy in Women with Recurrent Intrauterine Adhesions and Thin Endometrium
Source: Life (Basel). 2026 May 22;16(6):871. doi: 10.3390/life16060871 (PMC13302435; doi:10.3390/life16060871)
Supplement: Supplementary file 1 [file life-16-00871-s001.zip › life-4233892-supplementary.pdf]

Supplementary Materials:

Table S1. Morphological and immunohistochemical characteristics of the endometrium according to reproductive outcome.

Note: Data are presented as median (IQR) or n/N (%), as appropriate. P values indicate comparisons between the no live birth and live birth groups. Differences in denominators reflect the availability and adequacy of histological, immunohistochemical, and histochemical specimens for each analysis. **Me**, median; **IQR**, interquartile range; **HPF**, high power field; **MUM1**, multiple myeloma oncogene 1.

Parameter	Total (n=35)	No Live Birth (n=24)	Live Birth (n=11)	p-value
Histological findings				
Focal stromal fibrosis, n (%)	20/32 (62.5%)	15/21 (71.4%)	5 (45.5%)	0.18
Scattered lymphoid infiltration, n (%)	12 (34.3%)	9 (42.9%)	3 (27.3%)	0.424
Indifferent-type endometrium fragments, n (%)	4 (11.4%)	3 (14.3%)	1 (9.1%)	0.74
Edematous loose stroma, n (%)	2 (5.7%)	0 (0%)	2 (18.2%)	0.111
Endometrial atrophy, n (%)	1 (2.9%)	0 (0%)	1 (9.1%)	0.314
Immunohistochemical analysis (MUM1)				
MUM1-positive cases (>5 cells/10 HPF), n/N (%)	4/17 (23.5%)	3/13 (23.1%)	1/4 (25%)	0.271
MUM1-positive cells, Me (IQR)	0 (0;3)	0 (0;3)	2 (0.75;4.25) [n=4]	0.389
Collagen deposition (Heidenhain staining)				
Collagen area (mm²), Me (IQR)	0.05 (0;1.45)	0.05 (0;1.45)	0 (0;0.6)	0.542
Collagen-positive cases (>0 mm²), n/N (%)	13/27 (48.1%)	8/16 (50%)	5/11 (45.5%)	0.831
Collagen proportion (%), Me (IQR)	0 (0;12)	0.5 (0;15.5)	0 (0;1)	0.44
Collagen deposition (Weigert–van Gieson staining)				
Collagen area (mm²), Me (IQR)	0 (0;1.35)	0.05 (0;1.57)	0 (0;0.6)	0.488
Collagen-positive cases (>0 mm²), n/N (%)	13/27 (48.1%)	8/16 (50%)	5/11 (45.5%)	0.831
Collagen proportion (%), Me (IQR)	0 (0;13)	0.5 (0;19.25)	0 (0;1)	0.44

Data are presented as median (interquartile range) unless otherwise specified.
